# Gorlin and Goltz Syndrome: A Case Report with Surgical Review

**DOI:** 10.5005/jp-journals-10005-1199

**Published:** 2013-08-26

**Authors:** Rakesh Namdeoraoji Bahadure, Eesha Surendraji Jain, Gautam P Badole

**Affiliations:** Lecturer, Department of Pedodontics and Preventive Dentistry, Sharad Pawar Dental College, Wardha, Maharashtra, India; Postgraduate Student, Department of Pedodontics and Preventive Dentistry, FODS, Chhatrapati Shahuji Maharaj Medical University, Lucknow, Uttar Pradesh, India; Lecturer, Department of Conservative Dentistry, VSPM Dental College, Nagpur, Maharashtra, India

**Keywords:** Basal cell carcinoma, Gorlin syndrome, Nevoid basal cell carcinoma, Odontogenic keratocysts, Palmar/Plantar pits, Bifid rib, Internal strabismus

## Abstract

Gorlin and Goltz syndrome are a very complex syndrome and a multisystemic process that is characterized by the presence of multiple pigmented basocellular carcinomas, keratocysts in the jaws, palmar and/or plantar pits and calcification of the falx cerebri. Along with these major features a great number minor features have also been described which involves numerous skeletical, dermatology related, neurological, ophthalmological and reproductive anomalies. It exhibits high penetrance and variable expressivity. Presented here is the case of Gorlin-Goltz in a 12 years old male patient which was diagnosed through its oral and maxillofacial manifestations. Treatment of odontogenic keratocyst was done by enucleation without primary suturing. Iodoform dressing was kept to enhance the healing and to reduce the recurrence of the lesion.

It is important to provide the early diagnosis for detection of clinical and radiological manifestations in young patients and for provision of advice concerning preventive treatment like protection of the skin from the sunlight and genetic sensitivity testing so that possible complications associated with this syndrome can be prevented.

**How to cite this article:** Bahadure RN, Jain ES, Badole GP. Gorlin and Goltz Syndrome: A Case Report with Surgical Review. Int J Clin Pediatr Dent 2013;6(2):104-108.

## INTRODUCTION

Jaw cyst-basal cell nevus-bifid rib syndrome also known as a Basal cell nevus syndrome or Gorlin and Goltz syndrome. This syndrome was first described by Jarish and White in 1894, noticed the presence of multiple basocellular carcinoma. The incidence of this syndrome is estimated to be 1 in 50,000 to 1,50,000 in general population but may vary with region. The clinical features may be seen in first or second decade of life which is mainly diagnosed through its oral and maxillofacial manifestations. In 1960, Robert J Gorlin and Robert W Goltz^1^ established a classical triad that characterizes the diagnosis of this syndrome involving multiple basocellular epitheliomas, keratocysts in the jaws and bifid ribs. This triad was later modified by Rayner in 1977 who established that for giving the diagnosis at least cysts had to appear in combination with calcification of the falx cerebri or palmar and plantar pits.

Several reports have appeared in the medical literature describing this syndrome. Dental clinician should always be open to the possibility of encountering multiple lytic lesions of the jaws. Therapy may include various types of surgical procedures, often with high recurrence rate or some other early postoperative complication.

## CASE REPORT

**Fig. 1:**
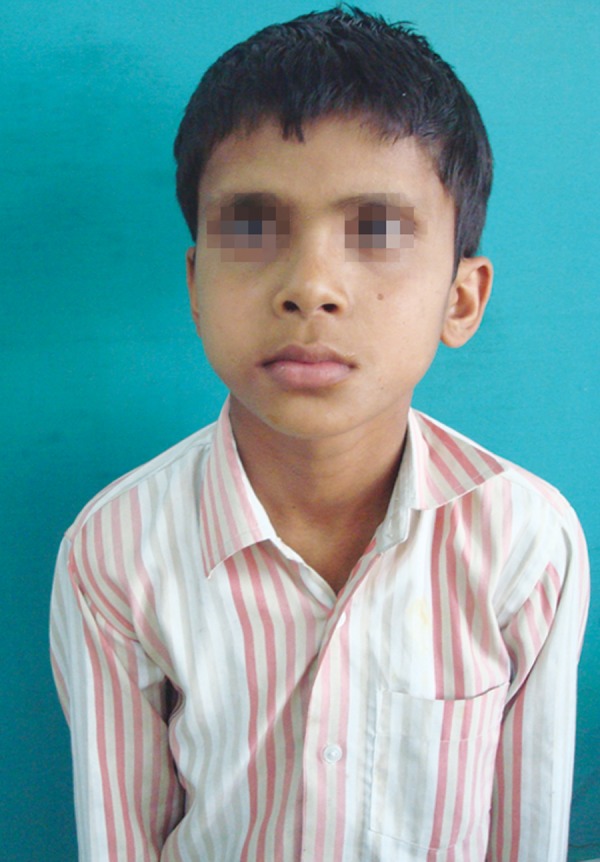
Swelling and internal strabismus in extraoral view

A male patient aged 12 years of age visited to the department of pedodontics and preventive dentistry with a chief complaint of swelling and pus discharge from the right side of the maxilla since 2 months. The growth of swelling was slow in nature and noticed when it was approximately 1 cm in size. When patient reported it was approximately 6 × 7 cm in diameter, extending from the canine to distal to the first permanent molar on right side of cheek (Fig. 1).

The swelling was also appeared on the right palate extending from the canine region to first permanent molar and upto the midline. The swelling was fluctuant on both sides and nontender. Numbness was noticed on the same side. The pus discharge from the 55 region was seen. The deciduous molars on the same side showed the grade III mobility.

**Fig. 2:**
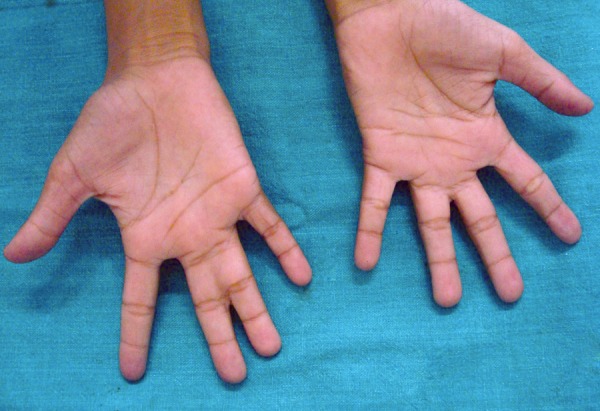
Palmar pits and webbing

General examination revealed a whitish child with altered gait, slurred speech, internal strabismus, head slanted on left side, occipitofrontal circumference was 63 cm (which was 52 cm in this age group), webbing on both hands and left leg. A slight elevated area seen on inspection on left side of chest which on palpation showed an tubercular filling. A small nevus seen on right side of upper trunk approximately 4 × 5 mm in dimension and it was noticed by parent that was increasing. Palmar pits were present which 14 in number on palms were. It was ranging from 2 × 2 mm to 0.3 × 0.5 mm (Fig. 2).

Medical, dental and family history was not significant. Routine laboratory tests were normal.

## RADIOGRAPHIC EXAMINATION

**Fig. 3:**
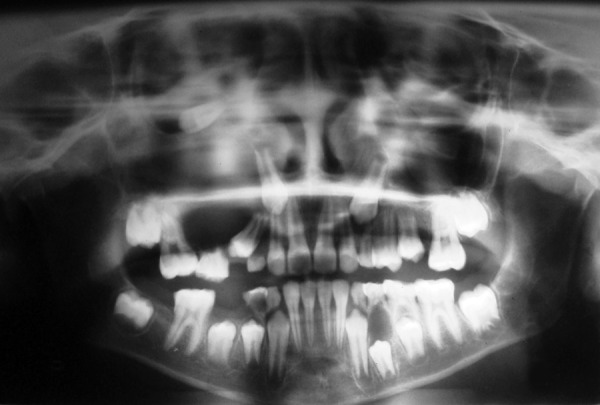
Panoramic view with multiple cyst

Orthopantomogram showed one lytic lesion in upper right side of jaw with unerupted 13 displaced from their normal position. Other lesion is seen in the mandible associated with unerupted 34 (Fig. 3).

**Fig. 4:**
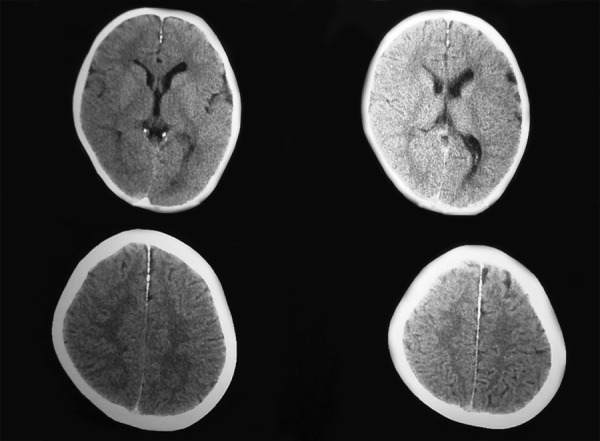
CT of mid face with cyst and nasal polyp

Coronal and axial computerized tomographic scan of skull showed the calcifications along the falx cerebri, tentorium cerebelli, dura over the convexity of left occipital lobe and in basal cisterns along the leptomeninges. Prominent bilateral lateral ventricles were seen (Fig. 4).

**Fig. 5:**
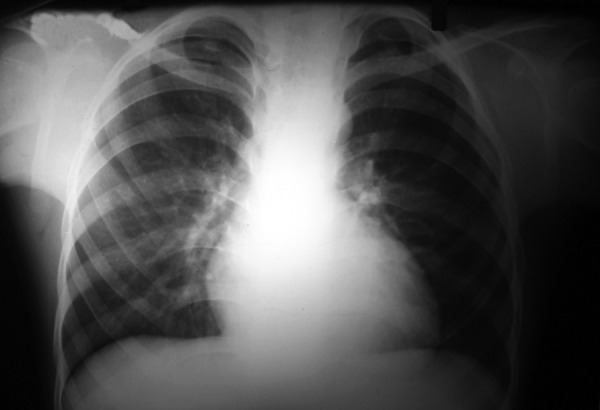
CT of skull with calcification

Chest X-ray showed the bilateral fourth bifid rib anteriorly (Fig. 5).

### Treatment Planning

**Fig. 6:**
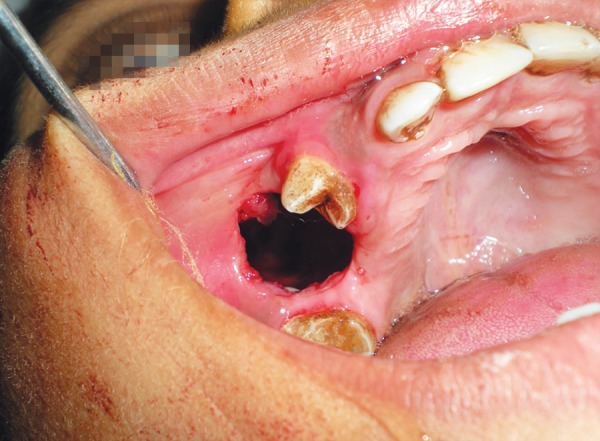
Fourth bifid ribs anteriorly

**Fig. 7:**
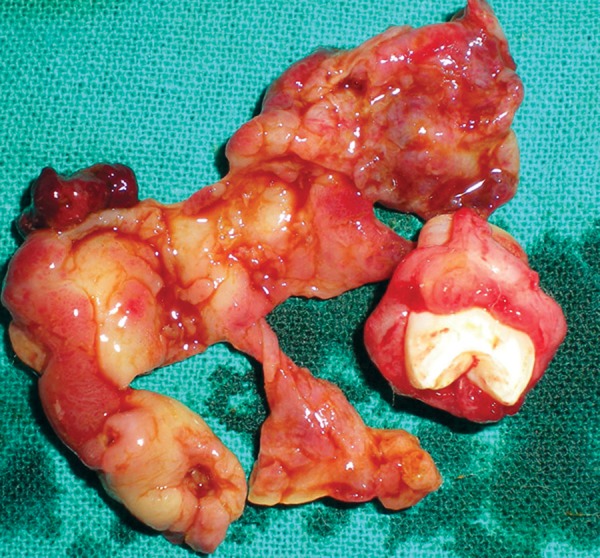
Enucleation of cyst without primary suturing

Marsupialization followed by enucleation of upper odontogenic keratocyst and scraping of bony cavity was done with surgical bur and curette. Iodoform dressing was given changing twice a week till the normal healing of cystic cavity. Specimen was sent for histopathological examination (Fig. 6 and 7).

The enucleation of cyst of lower jaw was done and scraping of bony cavity was done with surgical bur and curette. The specimen was sent for histopathological examination.

### Histopathological Examination

The report showed the parakeratinized stratified epithelium with an average thickness of 5 to 8 cells, with basal cells presents fenced up in a corrugated surface and a connective wall rich in mucopolisacaridos and with a variable number of microcysts and epithelial islets seen.

## DISCUSSION

It is an autosomal dominant inheritance disorder, probably a result of abnormalities in the patched tumor suppressor gene located on the chromosomes. 9q21-23^2,3^ PTCH gene present in Hedgehog receptor involve in embryonic development.^4^ Inactivity of Ptc or constitutive activity of Smo or Hh could lead to overactivity of Smo, resulting in neoplasm formation^3^ or molecular origin of OKC.^5^

The most important criteria to make a diagnosis for this syndrome are the presence of pigmented basocellular carcinomas, odontogenic keratocysts, palmar and/or plantar pits and ectopic calcifications of the falx cerebri.^6-10^ The diagnostic criteria for nevoid basal cell carcinoma was put forth by Evans et al and modified by Kimonis et al in 1997. According to him, diagnosis of Gorlin syndrome can be established when two major or one major and two minor criteria as described below are present.

Major criteria include:^11,12^

- More than two basal cell carcinoma or one basal cell carcinoma at younger than 30 years of age or more than 10 basal cell nevi
- Any odontogenic keratocyst (proven on histology) or polyostotic bone cyst
- Three or more palmar or plantar pits
- Ectopic calcification: Lamellar or early at younger than 20 years of age
- Falx cerebri calcification
- Positive family history of nevoid basal cell carcinoma.

Minor criteria include:^11,12^

- Congenital skeletal anomaly, bifid, fused, splayed, missing or bifid rib, wedged or fused vertebra
- Occipital-frontal circumference more than 97%
- Cardiac or ovarian fibroma
- Medulloblastoma
- Lymphomesentric cysts
- Congenital malformations such as cleft lip or palate, polydactylism or eye anomaly (cataract, coloboma, microphthalmus).

Other diagnostic findings in adults with nevoid basal cell carcinoma reported by Gorlin et al^13^ (1977) and their incidence of occurrences are:

### A. Skeletal Anomalies^11,13^

1. Bifid ribs, splayed/fused ribs, absent/rudimentary ribs (60-75%)–may be bilateral and several ribs may be affected
2. Scoliosis–seen in 30 to 40% of the patients
3. Hemivertebrae
4. Flame-shaped lucencies of hand/feet
5. Polydactyly
6. Syndactyly
7. Shortened 4th metacarpal.

### B. Craniofacial Anomalies^11,13^

1. Frontal bossing (25%): Increased size of calvaria (occipitofrontal circumference 60 cm or > in adults)
2. Brachycephaly
3. Macrocephaly (40%)
4. Coarse face (50%)
5. Calcification of the falxes (37-79%)
6. Tentorium cerebelli calcification
7. Bridged sella turcica
8. Heavy fused eyebrows
9. Broadened nasal root
10. Low positioning of occiput.

### B. Ophthalmic Findings

- Hypertelorism (40%)
- Glaucoma
- Exotropia choroidal and/or optic nerve coloboma
- Congenital amaurosis
- Congenital blindness and opaque cornea
- Ptosis, internal strabismus (15%) convergent/divergent, Chalazion
- Congenital or precocious cataract.

### C. Neurological Anomalies^11,13^

1. Agenesis/disgenesis of corpus callosum
2. Congenital hydrocephalus
3. Mental retardation
4. Medulloblastoma (3-5%) developing in the first 2 years of life. About 20% of them cause death during infancy
5. Meningioma (1% or <)
6. Schizoid personality.

### D. Oropharyngeal Anomalies^11,13^

Oral abnormalities are of fundamental importance mainly in childhood and adolescence and are important signs for diagnosis. They are:

1. Cleft lip/palate (4%)
2. High arched palate or prominent ridges (40%).

***Sexual Anomalies***

Uterine and ovarian fibromas (15%), supernumerary nipple Ovarian fibrosarcoma, hypogonadism and cryptorchidism.

***Calcified Ovarian Cysts***

Female distribution of the pubis hair, scarce beard in men and ginecomastia.

In the present case 3 major criterion as odontogenic keratocysts, palmar pits, falcine calcification and minor criteria as bifid rib, increase size of calvaria, scoliosis, internal strabismus, webbing of both hands and left leg, Sprengel scapular deformity were observed.

The odontogenic keratocysts represent from 3 to 15% out of the total number of odontogenic cysts^14^ and they appear in the 65 to 75% of the cases of the syndrome.^7,9-11^These cysts represent a particular entity that has been of interest, mainly due to biological aggressiveness and to the great amount of recurrence.^7,10,15^ Multiple odontogenic keratocysts, arising from the rests of dental lamina of the mandible and occasionally the maxilla are common in this disorder, with a peak incidence in the second and third decade of life. These are unilocular or multilocular, lined by stratified squamous epithelium and may contain displaced teeth. These cysts may be complicated by the development of pathological fractures, ameloblastomas and squamous cell carcinomas, and have a high rate of recurrence.^16,17^ The management of these lesions varies in aggressiveness from simple enucleation or curettage to ostectomy with curettage of the adjacent bone. In addition, the term ‘peripheral ostectomy’ has been used to describe an adjunctive surgical procedure following enucleation or curettage, in which the osseous walls of the defect are abraded with coarse surgical burs in order to ensure that any residual peripheral neoplastic tissue is removed.^18^ The obvious advantage of a conservative surgical choice is the preservation of the adjacent bone, soft tissue and dental structures. Reduced morbidity as well as a shorter hospital stay generally follows this course of therapy. Some large, destructive cases require segmental resection of the involved jaw bone with immediate or delayed reconstruction. All the cases required periodic clinical and radiological follow-up to check any early signs of recurrence.

Chemical cauterization is a proved adjunctive technique in case of odontogenic keratocysts and is useful to prevent recurrence by fixing the daughter cysts or remnants of epithelial lining that are not removed during the enucleation procedure. Carnoy's solution is a phenolic compound with tissue fixative properties. Voorsmit has demonstrated that Carnoy's solution penetrates the bone to a depth of 1.54 mm following a 5-minute application without any damage to the inferior alveolar nerve.^19^ In order to decide which technique must be employed, the following factors have to be taken into account: Lesion size, lesion extension, location, possible cortical and soft parts damage, the age and whether it is a primary or recurrent lesion.^6^ It is also important to detect if it is an isolated keratocyst or if it is associated with the syndrome, as Forsell et al^10^ have suggested the recurrence rate is of 63% in keratocysts associated to the syndrome and of 37% in the isolated ones. In present case scraping of cystic cavity with surgical bur and curette followed by iodoform dressing, changed twice a week till the cavity closure was done. It was used to reduce the recurrence and promote the healing of cystic cavity.

Patients suffering from the syndrome have to undergo checkups at least once a year, especially the ones having odontogenic keratocysts.^10^

Palmar and plantar pits are specific signs of this syndrome, and they appear as punctiform depressions in the palms and plants skin.^6,9^ These alterations are caused by the lack of a partial or complete absence of the corneal stratum. They are permanent, unpalpable and asymptomatic, with a depth ranging from 2 to 3 mm and a diameter that ranges from 1 to 3 mm.^6,9^ When the syndrome is present, they manifest in between the 50 and 70% of the patients and they usually get developed in the second decade of life, increasing in the number with the age and they can increase to 500.^11^

In the skull there is early onset of calcification occur with lamellar calcification of the falx cerebri 70 to 85%,^15^calcification of tentorium cerebelli 20%,^15^ and dura and choroids. Complete or partial bony bridging of the sella turcica due to calcification of the diaphragma sellae is seen in 25% of patients. Neurologic abnormalities include as cyst of the choroid plexus of the third and lateral ventricles, agenesis of the corpus callosum, glial nodules projecting from lateral ventricles congenital hydrocephalus, mental retardation, medulloblastomas and meningiomas. Ophthalmologic abnormalities seen are dystopia canthorum, internal strabismus, congenital blindness and hypertelorism.

Rib anomalies include splaying, synostosis, and bifid and cervical ribs. Unilateral or bilateral alterations in first to fourth ribs are most typical. Vertebral anomalies consist of block vertebrae, hemivertebrae, synostosis, spina bifida occulta and kyphoscoliosis. Shortening of the fourth metacarpal and small flame-shaped lucent areas in the phalanges and tubular bones of the arms have also been reported.

Patients with Gorlin Goltz syndrome require consistent sun protection. Genetic counseling that considers the genetic risks is advisable for all patients with this syndrome, both familial and sporadic.

Most of the time patients visit to the dental clinic with the complains of jaw swelling. Thus, responsibility of proper diagnosis and further treatment plan lies on the dental team. There are various treatment modalities that have been proposed for odontogenic keratocysts considering its recurrent nature and further complications. So, there should be a periodic follow-up at regular intervals of 6 months till 5 years followed by once annually for the entire life.
